# Supplementary figures and images for: Structure of Salmonella Flagellar Hook Reveals Intermolecular Domain Interactions for the Universal Joint Function
Source: Biomolecules. 2019 Sep 9;9(9):462. doi: 10.3390/biom9090462 (PMC6769732; doi:10.3390/biom9090462)

Supplementary file

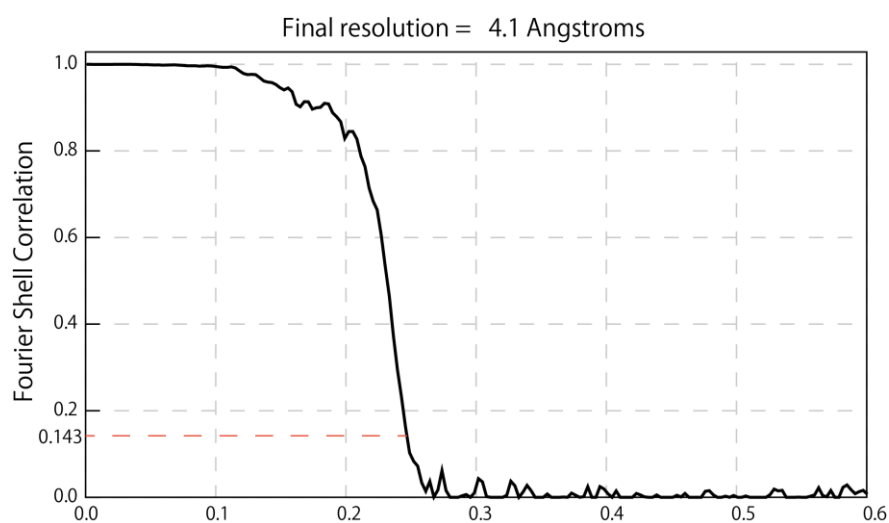

**Figure S1.** Fourier shell correlation of the 3D reconstruction.

Supplement: Supplementary file 1 [file biomolecules-09-00462-s001.pdf]
